# PDI-Mediated Reduction of Disulfide Bond on PSD95 Increases Spontaneous Seizure Activity by Regulating NR2A–PSD95 Interaction in Epileptic Rats Independent of *S*-Nitrosylation

**DOI:** 10.3390/ijms21062094

**Published:** 2020-03-18

**Authors:** Duk-Shin Lee, Ji-Eun Kim

**Affiliations:** Department of Anatomy and Neurobiology, Institute of Epilepsy Research, College of Medicine, Hallym University, Chuncheon 200-702, Korea; n84265@hallym.ac.kr

**Keywords:** epilepsy, nitric oxide, redox, seizure, siRNA, thiol

## Abstract

Postsynaptic density-95 (PSD95), a major scaffolding protein, is critical in coupling N-methyl-D-aspartate receptor (NMDAR) to cellular signaling networks in the central nervous system. A couple of cysteine residues in the N-terminus of PSD95 are potential sites for disulfide bonding, *S*-nitrosylation and/or palmitoylation. Protein disulfide isomerase (PDI) reduces disulfide bonds (S-S) to free thiol (-SH) on various proteins. However, the involvement of PDI in disulfide bond formation/*S*-nitrosylation of PSD95 and its role in epilepsy are still unknown. In the present study, acute seizure activity significantly increased the bindings of PDI to NR2A, but not to PSD95, while it decreased the NR2A–PSD95 binding. In addition, pilocarpine-induced seizures increased the amount of nitrosylated (SNO-) thiols, not total (free and SNO-) thiols, on PSD95. Unlike acute seizure, spontaneous seizing rats showed the increases in PDI–PSD95 binding, total- and SNO-thiol levels on PSD95, and NR2A–PSD95 interaction. PDI siRNA effectively reduced spontaneous seizure activity with decreases in total thiol level on PSD95 and NR2A–PSD95 association. These findings indicate that PDI-mediated reduction of disulfide-bond formations may facilitate the NR2A–PSD95 binding and contribute to spontaneous seizure generation in epileptic animals.

## 1. Introduction

The N-methyl-D-aspartate receptor (NMDAR) is one of the major excitatory receptors contributing to neurotransmission, synaptic plasticity and neuronal damage [1,2,3]. In addition, the NMDAR plays a key role in seizure generation and epileptogenesis [4,5,6]. NMDAR activity is regulated by redox modulation of disulfide bonds (S-S) and *S*-nitrosylation (-SNO) of cysteine residues on NR1 and NR2A subunits: Oxidation of free thiol (-SH) to disulfide bond and *S*-nitrosylation both inhibit NMDAR-evoked currents [7,8]. Recently, we have reported that protein disulfide isomerase (PDI), a member of the thioredoxin superfamily of redox proteins, increases seizure susceptibility via sulfhydration (reduction) of disulfide bonds (S-S to -SH + -SH) on NMDAR. Briefly, PDI knockdown or its neutralization prevents the induction of status epilepticus (SE, prolonged seizure activity) in response to pilocarpine (PILO) and inhibits spontaneous seizure activity in epileptic rats [5,9]. Therefore, the regulation of PDI activity is one of the potential therapeutic targets for epilepsy via NMDAR redox.

Postsynaptic density-95 (PSD95) is a major scaffolding protein that contains three PDZ domains, a SH3 (Src-homology-3) domain and a guanylate kinase domain [10]. PSD95 acts as an adapter molecule through protein–protein interactions mediated by the discrete domains. For example, the N-terminal of the first two PDZ domains of the PSD95 recognizes a four amino acid consensus sequence (-E-S/T-D-V) and allows it to bivalently link various ion channels, including NR2 subunits [11,12,13]. PSD95 is also required for NMDAR-mediated neuronal nitric oxide synthase (nNOS) activation [14]. Indeed, suppression of PSD95 expression inhibits NMDAR-mediated NOS activation and neuronal excitotoxicity [14]. However, synaptic NMDAR currents, subunit expression, localization and synaptic morphology remain unaltered in transgenic mice lacking PSD95, but they also shift the frequency dependence of NMDAR-mediated synaptic plasticity and impair spatial learning [15]. Thus, it is emphasized that PSD95 is critical in coupling NMDAR to cellular signaling networks and plays an important role in their biological functions within the central nervous system.

On the other hand, the N-terminus of PSD95 contains a pair of cysteine residue (cysteine 3 and 5 sites). These cysteine residues are potential sites for disulfide-bond formation, *S*-nitrosylation and/or palmitoylation [16,17]. In addition, disulfide linkage of each PSD95 protein in these sites mediates its multimerization as supramolecular aggregates [18] and facilitates receptor-clustering activity [19]. *S*-nitrosylation of these sites also influences PSD95 palmitoylation and its clustering at synapses [20]. Given the reductive role of PDI in disulfide bonds, it is interesting to evaluate whether PDI mediates disulfide-bond formation or *S*-nitrosylation on PSD95 during seizure generation. Here, we demonstrate that PILO-induced acute seizure activity significantly increased the bindings of PDI to NR2A, but not to PSD95, while it decreased the NR2A–PSD95. In addition, acute seizures increased the amount of *S*-nitrosylated (SNO)-thiols, but not total (free and SNO-) thiols, on PSD95. Unlike acute seizure models, spontaneously epileptic rats showed the increases in PDI–PSD95 binding, total- and SNO-thiol levels on PSD95, and NR2A–PSD95 interaction. PDI siRNA effectively reduced spontaneous seizure activity with the decreases in the total thiol level on PSD95 and NR2A–PSD95 association. Therefore, we suggest that PDI-mediated PSD95 redox may increase seizure susceptibility via facilitation of NR2A–PSD95 binding in epileptic animals.

## 2. Results

### 2.1. S-Nitrosylation and Disulfide Bond Formation on PSD95 in Acute Seizure Model

**Figure 1:**
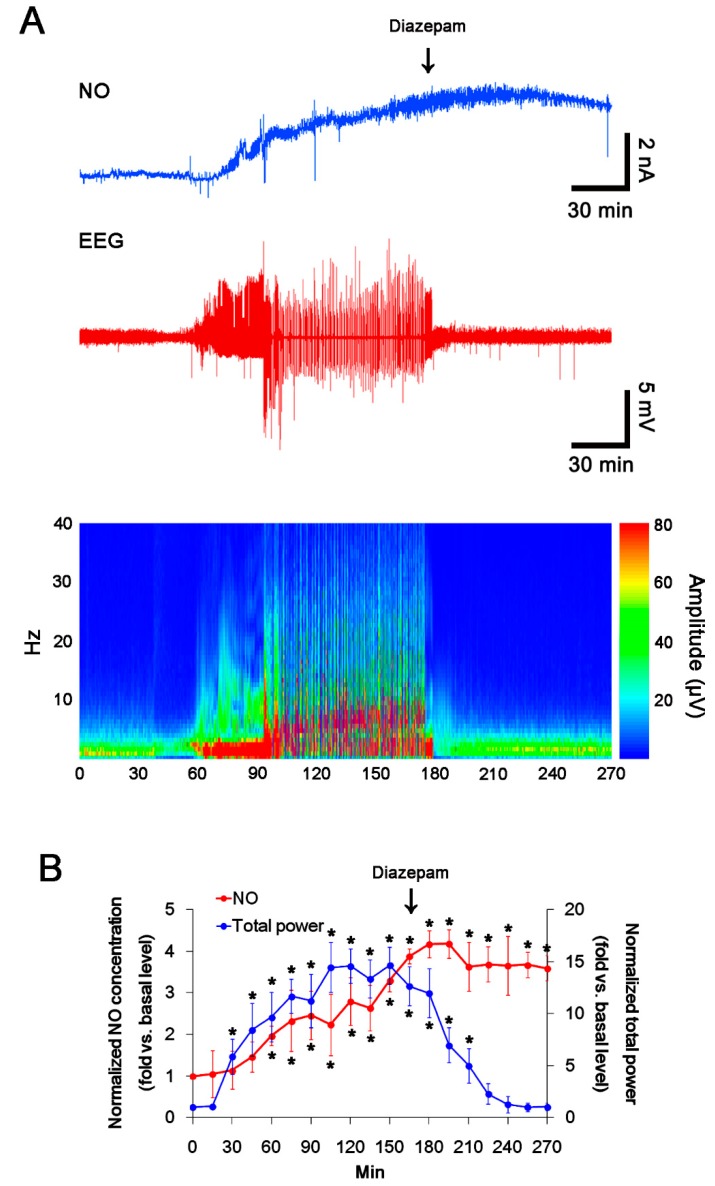
The real-time simultaneous monitoring of NO and EEG after PILO injection. NO level elevates after PILO injection and gradually increases during SE. Diazepam treatment recovers total EEG power to basal level, but not NO level. (**A**) Representative NO concentration (upper panel), EEG trace (middle panel) and frequency-power spectral temporal map (lower panel) in response to PILO. (**B**) Quantification of NO level and total EEG power in response to PILO (mean ± SEM; * *p* < 0.05 vs. basal level, repeated-measure one-way ANOVA; *n* = 7, respectively).

Consistent with our previous studies [21,22], the real-time simultaneous monitoring of NO and EEG revealed seizure onset and increase in NO level ~ 30 and ~ 60 min after PILO injection, respectively (*p* < 0.05 vs. basal level, repeated-measure one-way analysis of variance (ANOVA), *n* = 7; Figure 1A,B). NO concentration gradually increased during SE. Diazepam treatment effectively attenuated seizure activity to basal level, while it did not affect NO level (Figure 1A,B). Thus, our findings indicate that prolonged NO synthesis may be initiated by seizure onset, but may not be affected by seizure termination.

**Figure 2:**
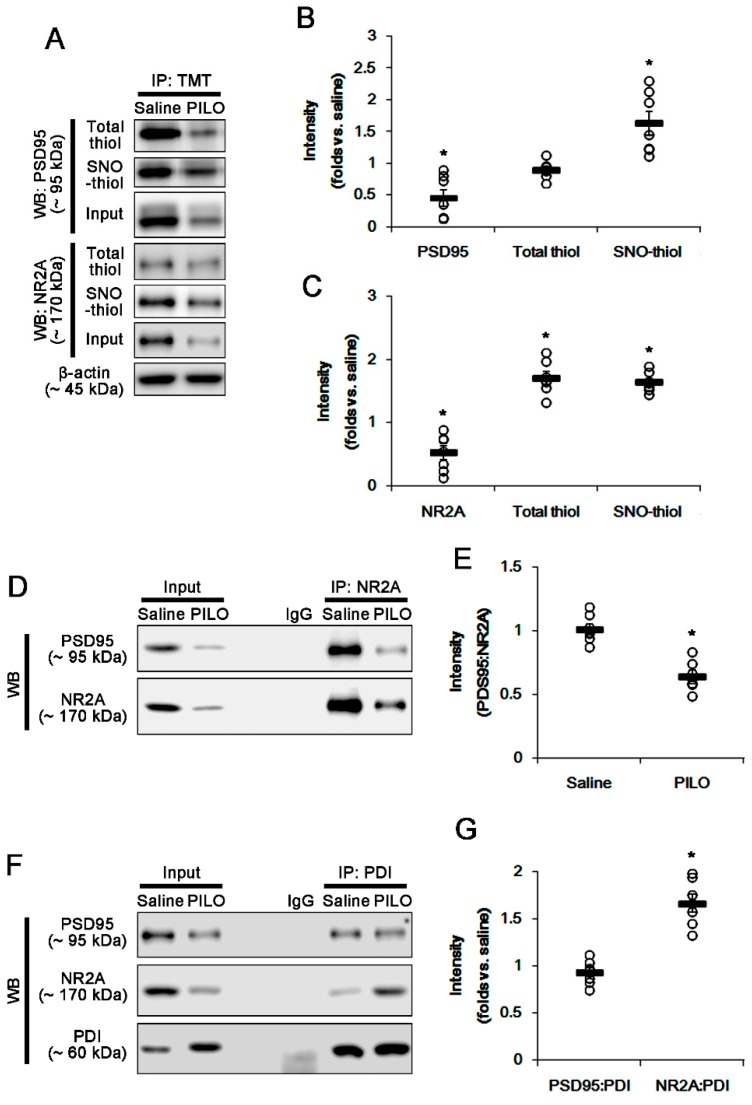
The effect of acute seizures on the total- and SNO-thiol levels on PSD95 and NR2A, and the bindings of NR2A–PSD95, PDI–PSD95 and PDI–NR2A in the hippocampus. (**A**) Representative Western blot for expression and the amounts of total- and SNO-thiols on PSD95 and NR2A. Acute seizures increase the number of SNO-thiols, but not total thiols, on PSD95. (**B**,**C**) Quantification of Western blot data. Open circles indicate each individual value. Horizontal bars indicate mean value. Error bars indicate SEM (* *p* < 0.05 vs. saline, Student’s *t*-test, *n* = 7, respectively). (**D**) Co-immunoprecipitation analysis of NR2A–PSD95 interaction following acute seizures. Acute seizure reduces both PSD95 and NR2A expression. However, the NR2A–PSD95 bindings are significantly reduced, as compared to saline-treated animals. (**E**) Quantification of Western blot data. Open circles indicate each individual value. Horizontal bars indicate mean value. Error bars indicate SEM (* *p* < 0.05 vs. saline, Student’s *t*-test, *n* = 7, respectively). (**F**) Co-immunoprecipitation analyses of PDI interactions with PSD95 and NR2A. Acute seizure increases PDI expression and PDI–NR2A binding, without altering PDI–PSD95 binding. (**G**) Quantification of Western blot data. Open circles indicate each individual value. Horizontal bars indicate mean value. Error bars indicate SEM (* *p* < 0.05 vs. saline, Student’s *t*-test, *n* = 7, respectively).

**Figure 3:**
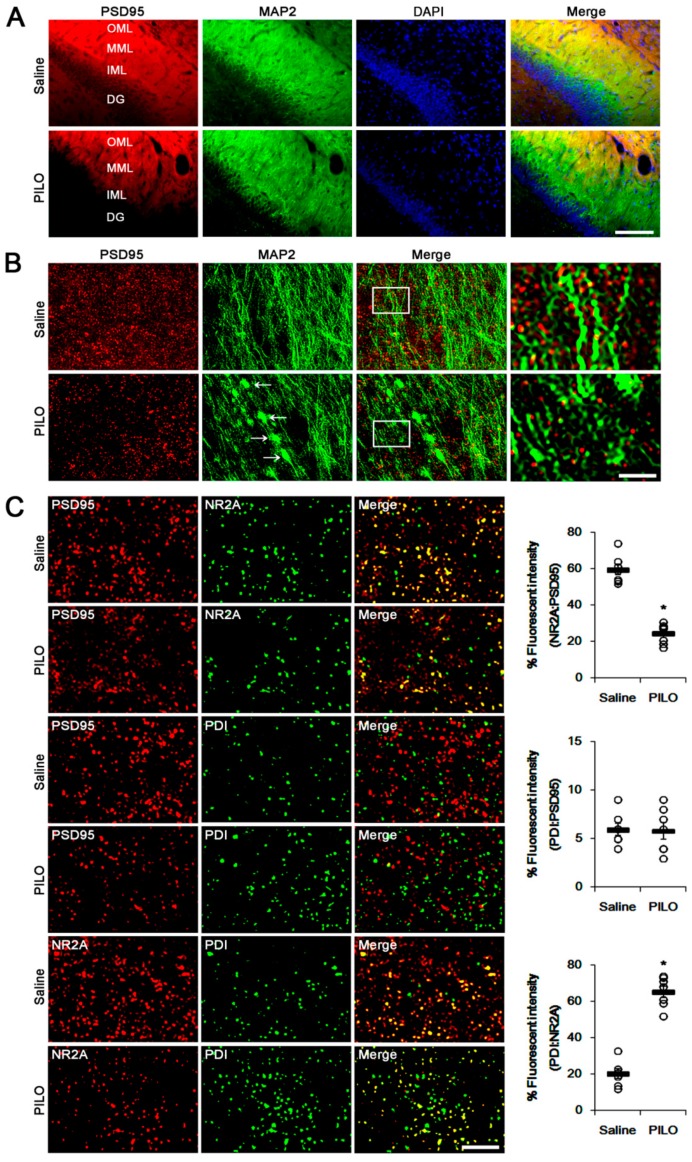
Representative double immunofluorescent photos for localization of PSD95, NR2A and PDI in the outer molecular layer of the upper blade of the dentate gyrus following acute seizures. (**A**) Low magnification photos of PSD95 and MAP2 in the upper blade of dentate gyrus. Bar = 50 μm. Abbreviations: OML, outer molecular layer; MML, middle molecular layer; IML, inner molecular layer; DG, dentate granule cell layer. (**B**) The reduced PSD95 puncta on MAP2-positive dendrites induced by acute seizures. Arrows indicate beading (damaging) dendrite induced by seizure activity. The right panels are high magnification images for rectangles. Bar = 10 (the left three panels) and 2.5 (the right panel) μm. (**C**) The changed colocalization of PSD95, NR2A and PDI in the molecular layer of the dentate gyrus. Following acute seizures, colocalization of NR2A:PSD95 is reduced. Acute seizure activity increases the colocalization of PDI:NR2A, while it does not affect colocalization of PDI:PSD95. Bar = 5 μm. The right panels indicate quantification of colocalized fluorescent intensity. Open circles indicate each individual value. Horizontal bars indicate mean value. Error bars indicate SEM (* *p* < 0.05 vs. saline, Student’s *t*-test, *n* = 7, respectively).

To investigate the effect of seizure activity on PSD95 redox and its *S*-nitrosylation, we measured total- and SNO-thiol levels on PSD95. PILO significantly reduced PSD95 expression (*p* < 0.05 vs. saline, Student’s *t*-test, *n* = 7; Figure 2A,B). With respect to activity-dependent downregulation of PSD95 expression [4,23], this phenomenon may be one of the adaptive responses for acute seizures. However, PILO increased the number of SNO-thiols, but not total thiols, on PSD95 (*p* < 0.05 vs. saline, Student’s *t*-test, *n* = 7; Figure 2A,B). Consistent with our previous study [5], PILO reduced NR2A expression (*p* < 0.05 vs. saline, Student’s *t*-test, *n* = 7; Figure 2A,C), but elevated the total- and SNO-thiol levels on NR2A (*p* < 0.05, Student’s *t*-test, *n* = 7; Figure 2A,C). PILO inhibited the NR2A–PSD95 binding (*p* < 0.05 vs. saline, Student’s *t*-test, *n* = 7; Figure 2D,E). However, PILO increased the PDI–NR2A binding, but not PDI–PSD95 co-assembly (*p* < 0.05 vs. saline, Student’s *t*-test, *n* = 7; Figure 2F,G). The immunofluorescent study revealed that PILO decreased PSD95 expression in the molecular layer of the dentate gyrus where the dendrites of dentate granule cells are localized (Figure 3A,B). PILO did not affect the colocalization of PDI within PSD95 puncta (Figure 3C). PILO decreased the colocalization of NR2A within PSD95 puncta, but increased colocalization of NR2A within PDI puncta (*p* < 0.05 vs. saline, Student’s *t*-test, *n* = 7, respectively; Figure 3C). Since PDI is a redox-active enzyme for NR2A [5], these findings indicate that PDI may participate in the reduction of disulfide bonds on NR2A, but not on PSD95, following acute seizures. In addition, increased NO concentration may nitrosylate free thiols on PSD95 and NR2A.

### 2.2. S-Nitrosylation and Disulfide Bond Formation on PSD95 in Epileptic Rats

**Figure 4:**
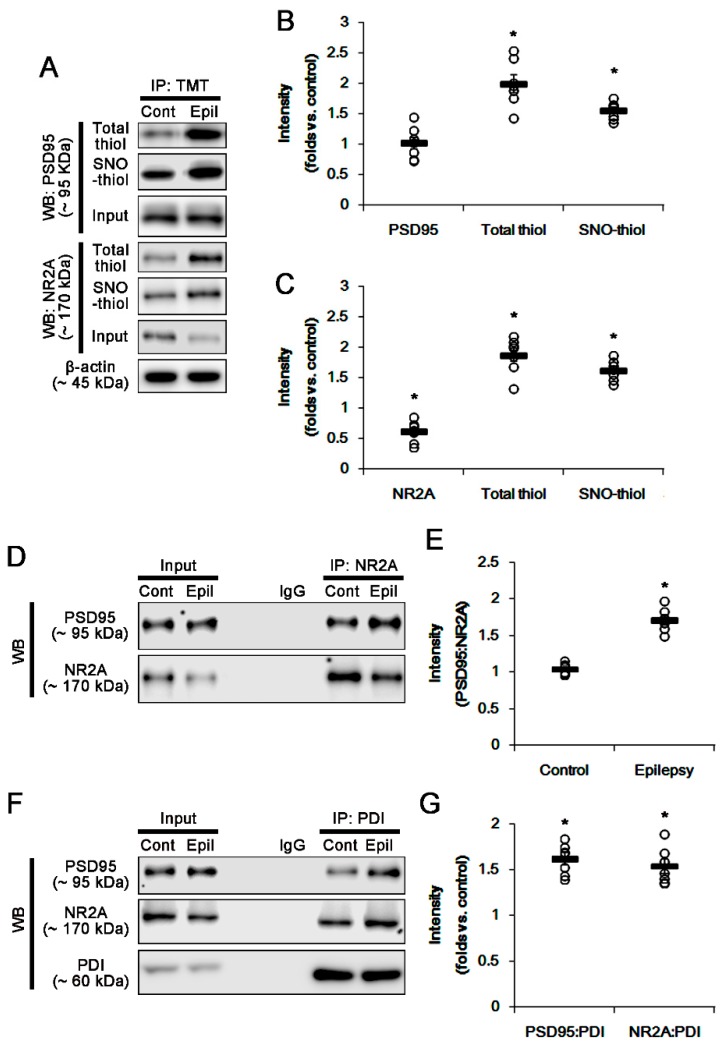
The amounts of total- and SNO-thiols on PSD95 and NR2A, and the bindings of NR2A–PSD95, PDI–PSD95 and PDI–NR2A in the hippocampus of epilepsy rats. (**A**) Representative Western blot for expression and the amounts of total- and SNO-thiol on PSD95 and NR2A. As compared to control animals, epilepsy rats show the increases in the total- and SNO-thiol levels on PSD95 without altered PSD95 expression. In addition, they show the increases in total- and SNO-thiol levels on NR2A, in spite of the reduced NR2A expression. (**B**,**C**) Quantification of Western blot data. Open circles indicate each individual value. Horizontal bars indicate mean value. Error bars indicate SEM (* *p* < 0.05 vs. saline, Student’s *t*-test, *n* = 7, respectively). (**D**) Co-immunoprecipitation analysis of NR2A–PSD95 interaction in epileptic rats. The NR2A–PSD95 binding is significantly increased, as compared to control animals. (**E**) Quantification of Western blot data. Open circles indicate each individual value. Horizontal bars indicate mean value. Error bars indicate SEM (* *p* < 0.05 vs. saline, Student’s *t*-test, *n* = 7, respectively). (**F**) Co-immunoprecipitation analyses of PDI–PSD95 and PDI–NR2A co-assemblies in epileptic rats. The bindings of PDI–PSD95 and PDI–NR2A are elevated in epileptic rats, as compared to control animals. (**G**) Quantification of Western blot data. Open circles indicate each individual value. Horizontal bars indicate mean value. Error bars indicate SEM (* *p* < 0.05 vs. saline, Student’s *t*-test, *n* = 7, respectively).

To evaluate whether PDI-mediated PSD95 redox is involved in spontaneous recurrent seizures, we explored the disulfide bonds and *S*-nitrosylation on PSD95 in chronic epileptic rats. The PSD95 expression level in epilepsy animals was similar to that observed in control (normal) animals. However, the ratio of total- and SNO-thiol levels on PSD95 was significantly increased in epileptic rats (*p* < 0.05 vs. control animals, *n* = 7; Figure 4A,B). Consistent with our previous studies [5], NR2A expression in epilepsy animals was lower than that in control animals. In contrast, the total- and SNO-thiol levels on NR2A were significantly increased in epileptic rats (*p* < 0.05 vs. control animals, Student’s *t*-test, *n* = 7; Figure 4A,C). The NR2A–PSD95 binding was also increased in epileptic rats (*p* < 0.05 vs. control animals, Student’s *t*-test, *n* = 7; Figure 4D,E). In addition, both PDI–PSD95 and PDI–NR2A co-assemblies were elevated in epileptic rats (*p* < 0.05 vs. control animals, Student’s *t*-test, *n* = 7; Figure 4F,G). These findings demonstrate that PDI may reduce disulfide bonds on PSD95 and NR2A in epileptic rats, unlike the acute seizure model.

### 2.3. Effect of PDI Knockdown on S-Nitrosylation and Disulfide-Bond Formation on PSD95

**Figure 5:**
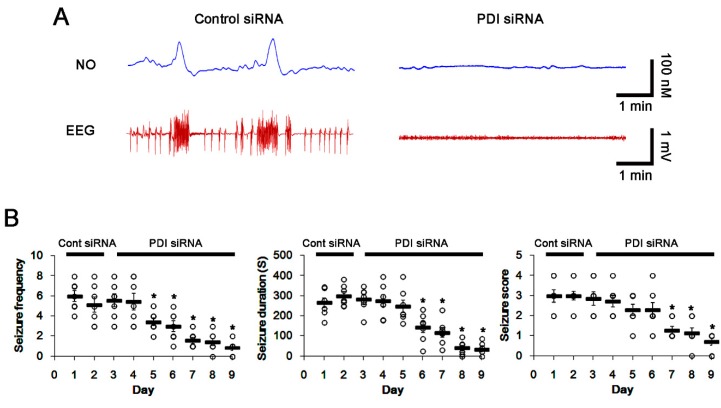
Effect of PDI knockdown on spontaneous seizure activity and NO generation in epilepsy. (**A**) Representative NO concentration and EEG trace for control siRNA-infused and PDI siRNA-infused epilepsy animals. In control siRNA-infused animals, NO level immediately rises after spontaneous seizure onset, and gradually decreases to basal level after seizure cessation (left panel). In PDI siRNA-infused animals, there is no alteration in NO level, due to the absence of spontaneous seizure activity (right panel). (**B**) Anticonvulsive effect of PDI siRNA on spontaneous seizure activity: the mean seizure frequency (left), seizure duration (middle) and behavioral seizure score (right). Open circles indicate each individual value. Horizontal bars indicate mean value. Error bars indicate SEM (* *p* < 0.05 vs. control siRNA, Kruskal–Wallis test with Dunn’s multiple comparison test, *n* = 7, respectively). PDI knockdown inhibits the spontaneous seizure activity in epileptic rats.

**Figure 6:**
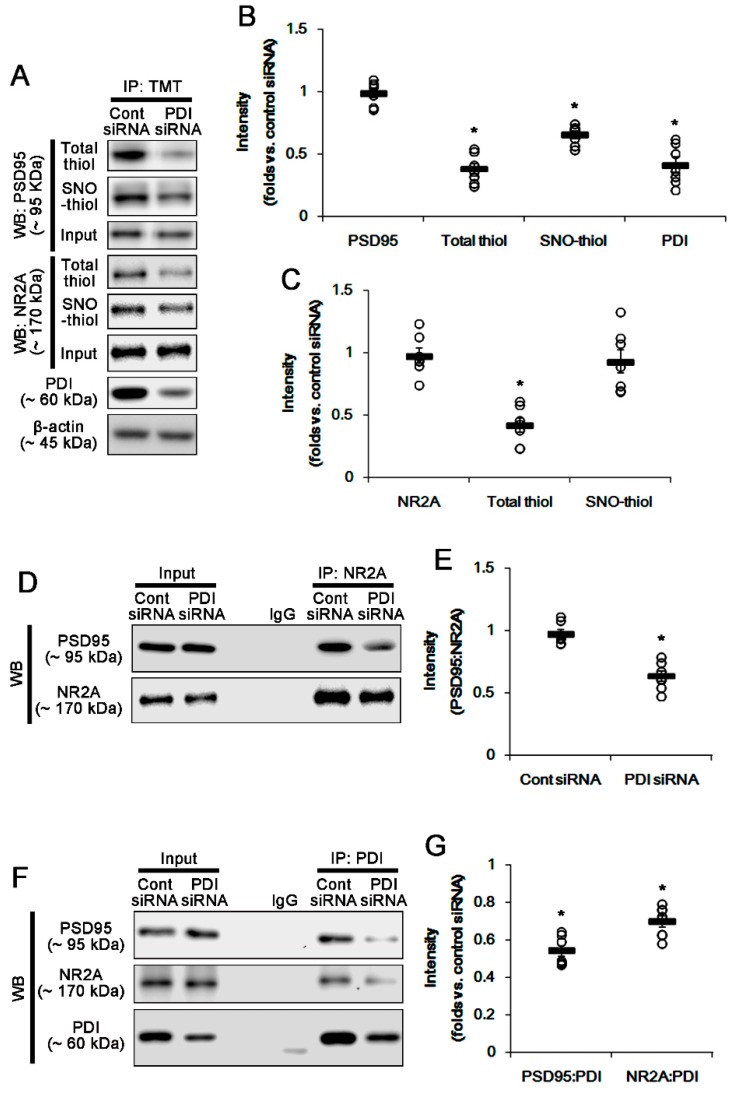
Effects of PDI knockdown on the amounts of total- and SNO-thiols on PSD95 and NR2A, and the bindings of NR2A–PSD95, PDI–PSD95 and PDI–NR2A in the hippocampus of epilepsy rats. (**A**) Representative Western blot for expression and the amounts of total- and SNO-thiols on PSD95. As compared to control siRNA, PDI siRNA reduces PDI protein level and the total thiol level on PSD95, without altering PSD95 expression. PDI siRNA also effectively decreases the total thiol level, but not SNO-thiol level, on NR2A in epilepsy rats. (**B**,**C**) Quantification of Western blot data. Open circles indicate each individual value. Horizontal bars indicate mean value. Error bars indicate SEM (* *p* < 0.05 vs. saline, Student’s *t*-test, *n* = 7, respectively). (**D**) Co-immunoprecipitation analysis of NR2A–PSD95 interaction in epileptic rats. The NR2A–PSD95 binding is significantly reduced by PDI knockdown. (**E**) Quantification of Western blot data. Open circles indicate each individual value. Horizontal bars indicate mean value. Error bars indicate SEM (* *p* < 0.05 vs. saline, Student’s *t*-test, *n* = 7, respectively). (**F**) Co-immunoprecipitation analyses of PDI–PSD95 and PDI–NR2A co-assemblies in epileptic rats. The PDI–PSD95 and PDI–NR2A bindings are diminished by PDI siRNA. (**G**) Quantification of Western blot data. Open circles indicate each individual value. Horizontal bars indicate mean value. Error bars indicate SEM (* *p* < 0.05 vs. saline, Student’s *t*-test, *n* = 7, respectively).

**Figure 7:**
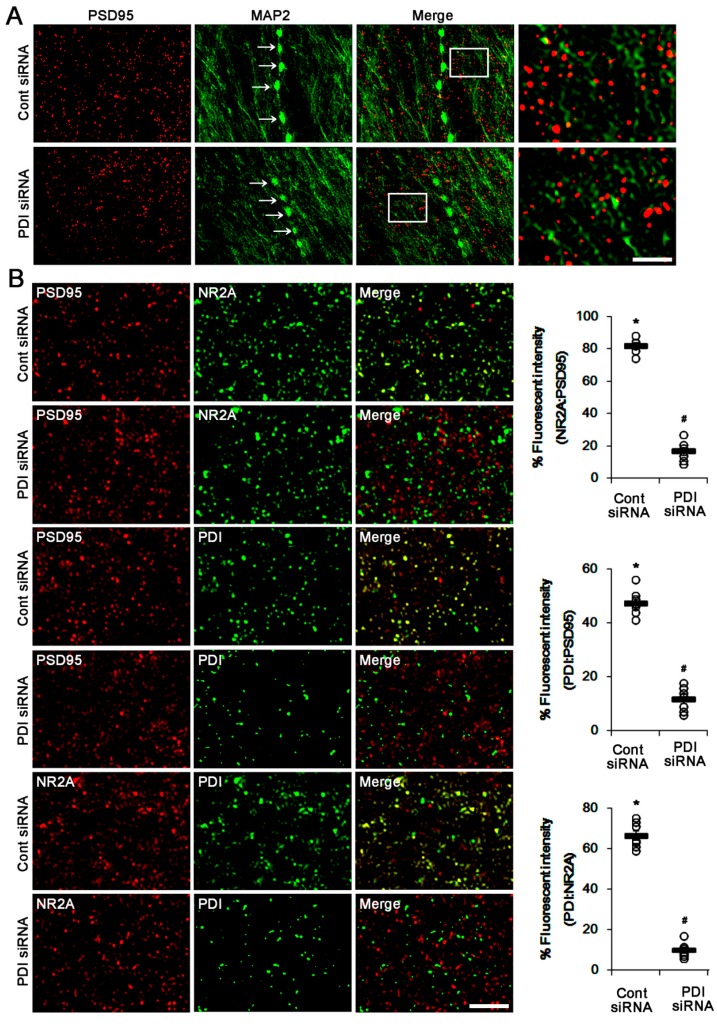
Effect of PDI knockdown on localization of PSD95, NR2A and PDI in the outer molecular layer of the upper blade of the dentate gyrus of epileptic rats. (**A**) PDI siRNA does not affect the density of PSD95 puncta on MAP2-positive dendrites in epileptic rats. Arrows indicate beading (damaging) dendrite induced by seizure activity. The right panels are high magnification images for rectangles. Bar = 10 and 2.5 μm. (**B**) The changed colocalization of PSD95, NR2A and PDI in the molecular layer of the dentate gyrus. As compared to control animals (see Figure 3B), colocalizations of NR2A:PSD95, PDI:PSD95 and PDI:NR2A are elevated in epileptic animals. PDI siRNA significantly reduced them, as compared to control siRNA. Bar = 5 μm. The right panels indicate quantification of colocalized fluorescent intensity. Open circles indicate each individual value. Horizontal bars indicate mean value. Error bars indicate SEM (*,# p < 0.05 vs. control animals and control siRNA, respectively, one-way ANOVA, *n* = 7, respectively).

To evaluate further the role of PDI–PSD95 interaction in spontaneous seizure activity, we applied PDI knockdown in epileptic rats. Similar to acute seizures, spontaneous seizure activities immediately raised the NO level. Consistent with our previous studies [5], PDI knockdown effectively reduced the spontaneous seizures in epileptic rats. Briefly, PDI siRNA reduced the mean seizure frequency and the total seizure duration from 6 to 0.86 (during 2 h recording session) and 267 to 33 s, respectively (*p* < 0.05 vs. control siRNA, Kruskal–Wallis test with Dunn’s multiple comparison test, *n* = 7; Figure 5A,B). PDI knockdown also decreased the behavioral seizure severity (Racine score) from 3 to 0.71 (*p* < 0.05 vs. control siRNA, Kruskal–Wallis test with Dunn’s multiple comparison test, *n* = 7; Figure 5A,B). PDI knockdown effectively declined the number of total thiols, but not SNO-thiols, on PSD95, without altering PSD95 protein level (*p* < 0.05 vs. control siRNA, Student’s *t*-test; Figure 6A,B). PDI siRNA also decreased the total thiol level on NR2A (*p* < 0.05 vs. control siRNA, Student’s *t*-test, *n* = 7; Figure 6A,C), while it did not affect the SNO-thiol level on NR2A and NR2A-protein level. Furthermore, PDI siRNA diminished the bindings of PDI–PSD95, PDI–NR2A and NR2A–PSD95 (*p* < 0.05 vs. control siRNA, Student’s *t*-test, *n* = 7; Figure 6D–G). The immunofluorescent study demonstrated that PDI knockdown did not affect PSD95 expression in the dendrites of dentate granule cells (Figure 7A). However, PDI siRNA decreased the colocalization of NR2A within PSD95 puncta, and those of PDI within PSD95 and NR2A puncta (*p* < 0.05 vs. control siRNA, one-way ANOVA, *n* = 7; Figure 7B). These findings indicate that PDI-mediated thiol formation on PSD95, rather than *S*-nitrosylation, may play an important role in ictogenesis via the increased NR2A–PSD95 bindings in epileptic rats.

## 3. Discussion

**Figure 8:**
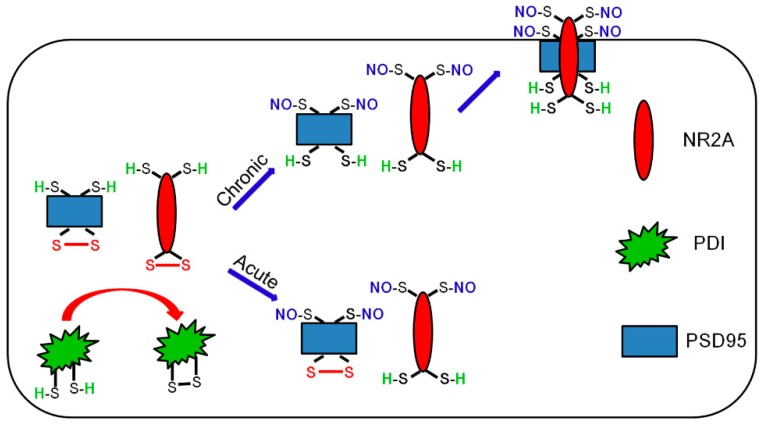
Schemes of the role of PDI in the seizure activity in acute and epilepsy model. PILO-induced acute seizure activity increases the bindings of PDI to NR2A, but not to PSD95, and total (free and SNO-) thiol level on NR2A. However, it decreases the NR2A binding to PSD95. Unlike acute seizure models, epileptic seizures increase PDI–PSD95 binding, total- and SNO-thiol levels on PSD95, and NR2A–PSD95 interaction. Since PDI siRNA effectively inhibits spontaneous seizure activity, our findings suggest that PDI-mediated PSD95 redox may increase seizure susceptibility via facilitation of NR2A–PSD95 binding in epileptic animals, independent of *S*-nitrosylation of PSD95.

The major findings in the present study are that PDI-mediated reduction of disulfide bonds on PSD95 may play an important role in the ictogenesis by the incorporation of NR2A in epileptic rats, independent of *S*-nitrosylation of PSD95 (Figure 8).

NMDAR overactivation leads to neuronal hyperexcitability [6]. Since oxidation and *S*-nitrosylation of cysteine residues on NMDAR subunits inhibit NMDAR-evoked currents [7,8], the regulation of redox status of NMDAR is one of the potential therapeutic anti-epileptic strategies [24,25,26]. Indeed, we have reported that PDI-mediated reduction of disulfide bonds on NMDAR, including NR2A, increases seizure susceptibility in acute-seizure and epilepsy models [5]. PSD95, a synaptic adapter protein [11,12,13], also has two N-terminal cysteines that are potential sites for disulfide bond formation and/or palmitoylation [16,17]. Thus, it is worth investigating whether PDI participates in redox status/*S*-nitrosylation of PSD95 and NR2A–PSD95 interaction. In the present study, acute seizures could not affect the total thiol level on PSD95. Unlike acute seizure model, epileptic animals showed the increases in PDI–PSD95 bindings, the total thiol level on PSD95 and NR2A–PSD95 co-assembly. Furthermore, the present data demonstrate that PDI knockdown in epilepsy rats effectively abolished these phenomena, accompanied by inhibitions of spontaneous seizure activity. Since cysteine residues are involved in intermolecular disulfide bond formation of PSD95 to organize clusters of ion channels [17], it is presumable that the PDI-mediated reduction in disulfide-bond formation would inhibit PSD95 multimerization and the PSD95-mediated NR2A clustering. However, PSD95 multimerization is not required for membrane association of PSD95 or other binding partners in the membrane [27]. Indeed, mutant mice lacking PSD95 show normal distribution and synaptic currents of NMDAR [15]. Thus, our findings suggest that PDI-mediated sulfhydration (reduction) of disulfide bonds on PSD95 may expose PDZ domains to increase the binding probability of PSD95 to NR2A in the epileptic hippocampus rather than the cell surface NR2A clustering.

The complex of PSD95 with NMDAR regulates neuronal activity, which is involved in modulating a range of synaptic functions and activities [14,15,28]. Suppression of PSD95 expression or inhibition of NMDAR–PSD95 bindings attenuates neuronal excitotoxicity [14,29]. However, there are many debates concerning the role of PSD95 in seizure activity. For example, the reduced NR2A–PSD95 bindings increase seizure susceptibility in response to pentylenetetrazol [30]. In contrast, in vitro seizure induction gradually enhances NR2A–PSD95 interaction [31]. In the present study, acute seizures decreased PSD95 and NR2A expressions accompanied by the reduction in the NR2A–PSD95 association. In epileptic rats, PSD95 level recovered to normal animal levels, while NR2A expression was lower than that in control animals. Furthermore, the NR2A–PSD95 binding was significantly increased, which was diminished by PDI knockdown. Since NR2A regulates channel open probability and drives synaptic strength [32,33], our findings indicate that the reduced NR2A–PSD95 interaction in acute seizure model may be an adaptive response for inhibiting excessive neuronal activity accompanied by the downregulation of both proteins. In contrast, the increased NR2A–PSD95 association in epileptic rats may play an important role in the spontaneous seizure generation. Indeed, PSD95 promotes Fyn-mediated tyrosine phosphorylation of NR2A, which potentiates NMDAR functionality [34]. Taken together, our findings provide the possibility that PDI-mediated reduction of disulfide bonds on PSD95 may increase NR2A–PSD95 binding and lead to spontaneous seizure activity via NMDAR hyperactivation in epileptic animals, concomitant with PDI-mediated reduction of NR2A [5].

Since N-terminal cysteines play additional roles for fatty acid modification (palmitoylation) of PSD95, *S*-nitrosylation on these cysteine residues competes with palmitoylation [17]. The enhanced excitatory input via glutamate receptors results in depalmitoylation of PSD95 in synapses [16], while the reduced synaptic activity increases PSD95 palmitoylation [34]. Interestingly, inhibition of palmitoylation increases *S*-nitrosylation of PSD95 and decreases NR2B–PSD95 binding [20]. In addition, *S*-nitrosylation facilitates further oxidation of thiols to disulfide bonds [8,35]. Therefore, it is likely that *S*-nitrosylation would be stable to reinforce the disulfide-bond formation on PSD95 and inhibit NR2A–PSD95 bindings. In the present study, acute seizures and spontaneous seizures in epileptic rats showed the increased SNO-thiol level on PSD95, accompanied by prolonged NO production. However, *S*-nitrosylation was not correlated to the amounts of disulfide bonds on PSD95, NR2A–PSD95 bindings and the PSD95 dendritic localizations. With respect to depalmitoylation and subsequent *S*-nitrosylation of PSD95 by the enhanced synaptic activity [13,15], our findings indicate that the upregulated *S*-nitrosylation of PSD95 may be a consequence from activity-dependent depalmitoylation of PSD95 for inhibition of seizure activity and may not have any influence on NR2A–PSD95 bindings.

In the present study, diazepam effectively attenuated PILO-induced seizure activity (total EEG power) to basal level, while it did not affect NO level. Thus, our findings indicate that prolonged NO synthesis may be initiated by seizure onset, but it may not be affected by seizure termination. Gualtieri et al. [36] have reported that diazepam cannot cease subtle convulsive movements (such as discontinuous clonic jerks of tongue, whiskers or eyelids, isolated or in combination, sometimes accompanied by tremors of the whole body), while it rapidly decreases the severity of motor seizures induced by PILO. In addition, diazepam cannot stop small-amplitude EEG activities, while it remarkably decreases the amplitude of epileptiform discharges induced by PILO. Therefore, it is likely that diazepam may convert from PILO-induced convulsive SE to non-convulsive SE. With respect to this previous report, it is considerable that the prolonged NO synthesis may be relevant to non-convulsive SE with continuous small amplitude EEG activities unaffected by diazepam.

The limitations of the present study are (1) the use of small-size animal groups (*n* = 7) and (2) the lack of clinical relevance. In the literature, the roles of PDI and NR2A–PSD95 bindings in seizure generation of human epilepsy patients and animal models have still to be determined. In patients, however, the increased co-assemblies of NR2B–PSD95 and NR1–PSD95 contribute to seizure generation [37]. Similar to the present study, the NR2A expression is downregulated in human cerebral heterotopia, accompanied by a less evident reduction of PSD95 proteins [38]. Furthermore, the brain PDI expression is increased in patients and in animal models of Lafora progressive myoclonus epilepsy (Lafora disease), which is a fatal autosomal recessive neurodegenerative disorder [39]. This PDI overexpression is speculated as an indicative of endoplasmic reticulum (ER) stress and an adaptive response to proteasome inhibition. Considering these previous reports, it is plausible that the upregulated PDI expression/activity may be involved in ictogenesis of Lafora disease patients, as well as other epileptic symptoms by increasing NR2A–PSD95 co-assembly, similar to the cases of NR1 and NR2B [37]. Further studies using a large size of animal groups and tissues obtained from epilepsy patients are needed to elucidate the role of PDI-mediated thiol formation on PSD95 in seizure activity.

On the other hand, therapeutic doses of NMDAR antagonists show the various adverse effects, although NMDAR inhibition is a potential target for epilepsy treatment [24,25,26]. In a previous study [5], we reported that PDI knockdown does not affect basal GABAergic or glutamatergic transmission without leading to ER stress in vivo. Thus, PDI-mediated regulations of NMDAR and/or PSD95 redox may be one of the therapeutic targets for epilepsy that does not have side effects.

## 4. Materials and Methods

### 4.1. Experimental Animals and Chemicals

Male Sprague Dawley (SD) rats (7 weeks old) were used in the present study. All experimental protocols described below were approved by the Institutional Animal Care and Use Committee of Hallym University (Chuncheon, South Korea, Hallym R1-2013-107, 3rd December 2015, and Hallym 2018-3, 30th April 2018). All efforts were made to minimize animal suffering. All reagents were obtained from Sigma-Aldrich (St. Louis, MO, USA), except as noted.

### 4.2. Acute Seizure Model

As described previously [5,40,41], we generated an acute seizure model induced by PILO. Briefly, animals were anesthetized (urethane, 1.5 g/kg i.p.) and placed in a stereotaxic frame. Recording electrode (Plastics One Inc., Roanoke, VA, USA) and NO sensor (ISO-NOPF200-L10, World Precision Instruments, Sarasota, FL, USA) were implanted into the left and right dorsal hippocampus (3.8 mm posterior; 2.0 mm lateral; 2.6 mm depth from bregma), respectively. The reference electrode was placed in the posterior cranium, over the cerebellum. After recording a stable baseline for at least 30 min, animals were given PILO (380 mg/kg i.p.) 20 min after atropine methylbromide (5 mg/kg i.p.). Total power and NO concentration were measured during the 270-minute recording session from each animal, with a DAM 80 differential amplifier (0.1–3000 Hz bandpass; World Precision Instruments, Sarasota, FL, USA) and Free radical analyzer (TBR4100, World Precision Instruments, Sarasota, FL, USA). Two hours after seizure onset, diazepam (Valium; Hoffman la Roche, Neuilly sur-Seine, France; 10 mg/kg, i.p.) was administered. The data were analyzed by using LabChart Pro v7 software (AD Instruments, Bella Vista, New South Wales, Australia). EEG and NO level were also measured in some epileptic rats, with the same method, without atropine methylbromide, PILO and diazepam treatments. After recording, animals were used for Western blot, co-immunoprecipitation, measurements of thiols and *S*-nitrosylation or immunohistochemistry (see below).

### 4.3. Epileptic Rat Model

SE was induced by intraperitoneal injection of PILO. Rats were treated with PILO (380 mg/kg i.p.) 20 min after atropine methylbromide (5 mg/kg i.p.). Control animals received an equal volume of normal saline instead of PILO after the pretreatment with atropine methylbromide. Diazepam (Valium; Hoffman la Roche, Neuilly sur-Seine, France; 10 mg/kg, i.p.) was administered 2 h after onset of SE and repeated, as needed. Animals were video-monitored 8 h a day, for general behavior and occurrence of spontaneous seizures by 6 weeks after SE. Rats showing spontaneous recurrent seizures were used as epileptic animals [5,41,42].

### 4.4. PDI Knockdown and Analysis of Chronic Seizure Activity

We applied a modified protocol for the effect of PDI knockdown on spontaneous seizure activity in epileptic rats, based on our previous reports [5,42]. Epileptic rats were anesthetized with Isoflurane (3% induction, 1.5%–2% for surgery and 1.5% maintenance in a 65:35 mixture of N2O:O2). A brain infusion kit 1 (Alzet, Cupertino, CA, USA) was implanted into the right lateral ventricle (1 mm posterior; 1.5 mm lateral; 3.5 mm depth from bregma) and connected to an osmotic pump (1007D, Alzet, USA) containing (1) control siRNA and (2) PDI siRNA, respectively. A PDI siRNA sequence corresponding to coding region (5′→3′) is sense: CUGCAAAACUGAAGGCAGAUU, and antisense: UCUGCCUUCAGUUUUGCAGUU. A non-silencing RNA was used as the control siRNA. The osmotic pump was subcutaneously placed in the interscapular region. Some animals were also implanted by monopolar stainless-steel electrode (Plastics One, Roanoke, VA, USA) implanted into the left dorsal hippocampus (3.8 mm posterior; 2.0 mm lateral; 2.6 mm depth from bregma). After baseline seizure activity (control siRNA treatment) was determined over 2 days, *PDI* siRNA was administered over a 7-day period. Between trials, the minipump was changed out under Isoflurane anesthesia. Every day, each animal was observed by video-EEG monitoring (2 h/day) at the same time. EEG analysis was performed by LabChart Pro v7 (AD Instruments, Bella Vista, New South Wales, Australia). Behavioral seizure severity was evaluated according to Racine′s scale: (1) immobility, eye closure, twitching of vibrissae, sniffing and facial clonus; (2) head nodding associated with more severe facial clonus; (3) clonus of one forelimb; (4) rearing, often accompanied by bilateral forelimb clonus; and (5) rearing with loss of balance and falling accompanied by generalized clonic seizures. After recording, animals were used for Western blot, co-immunoprecipitation, measurements of thiols and *S*-nitrosylation or immunohistochemistry (see below).

### 4.5. Western Blot

Under urethane anesthesia (1.5 g/kg, i.p.), animals were sacrificed by decapitation. Thereafter, the hippocampus was dissected out and homogenized in lysis buffer (50 mM Tris containing 50 mM 4-(2-hydroxyethyl)-1-piperazineethanesulfonic acid (pH 7.4), ethylene glycol tetraacetic acid (pH 8.0), 0.2% Tergitol type NP-40, 10 mM ethylenediaminetetraacetic acid (pH 8.0), 15 mM sodium pyrophosphate, 100 mM β-glycerophosphate, 50 mM NaF, 150 mM NaCl, 2 mM sodium orthovanadate, 1 mM phenylmethylsulfonyl fluoride and 1 mM dithiothreitol). Total protein content was measured by BCA protein assay kit. Western blotting was performed according to standard procedures. The primary antibodies were mouse anti-PDI (1:1000, #ab2792, Abcam, Discovery Drive, Cambridge Biomedical Campus, Cambridge, UK), rabbit anti-PSD95 (1:500, #ab18258, Abcam, Discovery Drive, Cambridge Biomedical Campus, Cambridge, UK) and rabbit anti-NR2A (1:1000, #OPA1-04021, ThermoFisher, Waltham, MA, USA). The rabbit anti-β-actin primary antibody (1:6000, #A5316, Sigma, St. Louis, MO, USA) was used as internal reference. The signals were scanned and analyzed by ImageQuant LAS4000 system (GE Healthcare Korea, Seoul, South Korea). The values of each sample were normalized with the corresponding amount of β-actin.

### 4.6. Co-Immunoprecipitation

The hippocampal tissues were lysed in radioimmune precipitation buffer (RIPA) with protease and phosphatase inhibitor cocktails (Roche Applied Sciences, Penzberg, Upper Bavaria, Germany) and 1 mM sodium orthovanadate. After calibration of total protein concentrations, equal amounts of proteins were precipitated with the primary antibody and subsequent protein G sepharose, at 4 °C, overnight [5]. Beads were collected, eluted in sample buffer and boiled at 95 °C for 5 min. Next, Western blotting was performed according to standard procedures.

### 4.7. Measurement of Total- and SNO-Thiols

Modified biotin switch assay was performed with the *S*-nitrosylation Western Blot Kit (ThermoFisher, Waltham, MA, USA), according to the manufacturer’s protocol. Briefly, lysates were reacted with ascorbate in HENS buffer for specific labeling with iodoTMTzero reagents with MMT pretreatment (SNO-thiol) or not (total thiol). Protein labeling can be confirmed by Western blot, using TMT antibody. Thereafter, TMT-labeled proteins were purified by Anti-TMT Resin, eluted by TMT elusion buffer and identified by Western blot, according to standard procedures.

### 4.8. Immunohistochemistry

Rats were anesthetized with urethane anesthesia (1.5 g/kg, i.p.) and perfused transcardially with 4% paraformaldehyde in 0.1 M phosphate buffer (PB, pH 7.4). Brains were post-fixed in the same fixative overnight and then cryoprotected and sectioned at 30 μm with a cryostat. Free-floating coronal sections were incubated in PDI antibody in PBS containing 0.3% Triton X-100, overnight, at room temperature. Tissue sections were incubated with a mixture of mouse anti-MAP2 (1:200, #MAB3418, Millipore, Burlington, MA, USA)/rabbit anti-PSD95 (1:500, #ab18258, Abcam, Discovery Drive, Cambridge Biomedical Campus, Cambridge, UK), rabbit anti-NR2A (1:1000, #OPA1-04021, ThermoFisher, Waltham, MA, USA)/mouse anti-PDI (1:1000, #ab2792, Abcam, Discovery Drive, Cambridge Biomedical Campus, Cambridge, UK), rabbit anti-PSD95 (1:500, #ab18258, Abcam, Discovery Drive, Cambridge Biomedical Campus, Cambridge, UK)/mouse anti-PDI (1:1000, #ab2792, Abcam, Discovery Drive, Cambridge Biomedical Campus, Cambridge, UK) or rabbit anti-NR2A (1:1000, #OPA1-04021, ThermoFisher, Waltham, MA, USA)/mouse anti-PSD95 (1:100, #MAB1596, Millipore, Burlington, MA, USA) antibodies in PBS containing 0.3% Triton X-100, overnight, at room temperature. Thereafter, sections were visualized with Cy2- and Cy3-conjugated secondary antibody. Immunoreaction was observed and analyzed, using an Axio Scope microscope (Carl Zeiss Korea, Seoul, South Korea) or a confocal laser scanning microscope (LSM 710, Carl Zeiss Korea, Seoul, South Korea). To establish the specificity of the immunostaining, a negative control test was carried out with pre-immune serum instead of the primary antibody. No immunoreactivity was observed for the negative control in any structures. All experimental procedures in this study were performed under the same conditions and in parallel [5].

### 4.9. Statistical Analysis

Quantitative data are expressed as mean ± standard error of the mean. After evaluating the values on normality by using the Shapiro–Wilk *W*-test, data were analyzed by Student’s *t*-test, repeated-measure one-way analysis of variance (ANOVA), one-way ANOVA with Bonferroni’s post hoc test for multiple comparisons, and Kruskal–Wallis test with Dunn’s multiple comparison test. A *p* < 0.05 is considered statistically different.

## 5. Conclusions

The present data show that PDI-mediated reduction of disulfide bond formations on PSD95 facilitated the NR2A–PSD95 binding, which is critical for spontaneous seizure generation in epileptic animals (Figure 8). However, *S*-nitrosylation of PSD95 may not be involved in seizure onset or cessation. Therefore, these findings suggest that oxidation of PSD95 may be a potential anti-epileptic therapeutic target more than *S*-nitrosylation.
